# Temporal Decay in Timber Species Composition and Value in Amazonian Logging Concessions

**DOI:** 10.1371/journal.pone.0159035

**Published:** 2016-07-13

**Authors:** Vanessa A. Richardson, Carlos A. Peres

**Affiliations:** Centre for Ecology, Evolution and Conservation, School of Environmental Sciences, University of East Anglia, Norwich, NR4 7TJ, United Kingdom; University of Vermont, UNITED STATES

## Abstract

Throughout human history, slow-renewal biological resource populations have been predictably overexploited, often to the point of economic extinction. We assess whether and how this has occurred with timber resources in the Brazilian Amazon. The asynchronous advance of industrial-scale logging frontiers has left regional-scale forest landscapes with varying histories of logging. Initial harvests in unlogged forests can be highly selective, targeting slow-growing, high-grade, shade-tolerant hardwood species, while later harvests tend to focus on fast-growing, light-wooded, long-lived pioneer trees. Brazil accounts for 85% of all native neotropical forest roundlog production, and the State of Pará for almost half of all timber production in Brazilian Amazonia, the largest old-growth tropical timber reserve controlled by any country. Yet the degree to which timber harvests beyond the first-cut can be financially profitable or demographically sustainable remains poorly understood. Here, we use data on legally planned logging of ~17.3 million cubic meters of timber across 314 species extracted from 824 authorized harvest areas in private and community-owned forests, 446 of which reported volumetric composition data by timber species. We document patterns of timber extraction by volume, species composition, and monetary value along aging eastern Amazonian logging frontiers, which are then explained on the basis of historical and environmental variables. Generalized linear models indicate that relatively recent logging operations farthest from heavy-traffic roads are the most selective, concentrating gross revenues on few high-value species. We find no evidence that the post-logging timber species composition and total value of forest stands recovers beyond the first-cut, suggesting that the commercially most valuable timber species become predictably rare or economically extinct in old logging frontiers. In avoiding even more destructive land-use patterns, managing yields of selectively-logged forests is crucial for the long-term integrity of forest biodiversity and financial viability of local industries. The logging history of eastern Amazonian old-growth forests likely mirrors unsustainable patterns of timber depletion over time in Brazil and other tropical countries.

## Introduction

Biological populations with slow life-histories and yielding commercially valuable natural resources have been predictably overexploited over the course of human history. This often occurs through a ratchet effect whereby a beneficiary is influenced by the previous highest level of offtake of a given resource, stemming from heavily subsidised industries to the point of economic extinction or demographic collapse [1,2]. This effect may also apply to extractive industries fuelled by non-renewable resources as illustrated by peak oil [3] and peak phosphorous [4]. Yet overexploitation is not necessarily inevitable but rather a common consequence of low-governance and open-access systems or ‘the tragedy of the commons’ [5,6]. Emblematic historical collapses of myriad wild resource populations include timber species [7] and non-timber forest species such as palm heart [8], or the Antarctic blue whale, cod stocks off New England and eastern Canada, and Mediterranean bluefin tuna [9–11]. Over 230 overharvested fish populations have shown median reductions of 83% from known historical levels [12], often within a mere 15 years of exploitation [13].

Economic extinction risk in terrestrial resource populations is analogous to those in marine ecosystems [7,14]. For centuries, old-growth timber stocks have been rapidly mined at the expense of future cohorts [15]. The once abundant endemic stands of Brazil-wood or Pau Brasil (*Caesalpinia echinata*, Leguminosae-Mimosoidae), a colonial source of red dye and hardwood after which Brazil was named, were heavily harvested four centuries ago to the brink of extinction, except for a few small, highly inbred relict populations [16]. Overexploitation of tropical forest tree species remains ubiquitous today, targeting prime timber and nontimber resources such as big-leaf mahogany (*Swietenia macrophylla* [17]) and Brazil-nuts (*Bertholletia excelsa* [18]). Amazonian rosewood populations deriving highly prized linalol essential oils (*Dalbergia nigra* and *Aniba rosaeodora*) have also been severely depleted by the high-end perfume industry to the point of widespread extinctions [19].

Whilst logging operations in tropical forests are highly variable in the degree to which they can be defined as sustainable, international consensus still deems logging to be one of the best compromises between land-use revenue and forest conservation [20], and timber to be an inherently renewable resource [21]. However, clear signs of ‘peak timber’ are already evident in Asian markets, as the region fast approaches a typical symmetric ‘Hubbert Curve’ logistic distribution seen in many overexploited non-renewable resources [22]. Counterintuitively, harvest levels that may be optimal from the perspective of individual harvesters may lead to elimination of the resource population [7,10]. Multi-temporal studies indicate that high individual discount rates can encourage the liquidation of commercially valuable timber stocks even when land-tenure is secure, wherever these can provide additional short-term revenues. These can be banked or reinvested into more profitable land-use options, rather than allowing longer time-horizons of slow timber regrowth in regenerating stands [23–25]. This often results in a highly degraded natural resource capital, with few options for alternative extractive industries. For instance, selectively logged Amazonian forests are much more likely to be deforested than unlogged forests [26], and local livelihoods generally follows a typical boom-and-bust trajectory at selectively-logged and subsequently deforested development frontiers [27] (but see [28]).

Sequential harvests in old-growth tropical forests typically progress from high-value, shade-tolerant, long-lived and large-girthed tree species (with generally high wood density, and often described as hard or heavy-wooded) toward a greater reliance on short-lived, low-value pioneer species (low wood density, or light-wooded) [29], with over 300 tree species considered to be commercially valuable in eastern Amazonia [30]. Valuable Amazonian timber tree species vulnerable to moderate and high extinction-risk include a range of canopy and emergent species such as big-leaf mahogany, Brazil-nut tree, ipê (*Tabebuia serratifolia*, *T*. *impetiginosa*), jatobá (*Hymenaea courbaril*), freijó cinza (*Cordia goeldiana*) and pau amarelo (*Euxylophora paraensis*) [30,31]. This may result in a predictable compositional shift from heavy-wooded to light-wooded species following each cutting cycle [32]. Market dynamics change to reflect this trend. In the 1990s, new logging frontiers in Peruvian Amazonia were highly selective targeting mahogany and up to 80–90 species [24], but 350 timber species were already being harvested in eastern Amazonia in the same decade [30]. Industrial scale logging is highly selective in Central-West Africa, with 95% of the offtake from the Congo basin comprising only 55 species [33]. Notwithstanding ambiguities with species identification and nomenclature, a few hundred timber species are still currently available in Amazonian markets [34] from the approximately 16,000 described and undescribed species in the Amazonian tree flora [35]. Compositional profiles of species selectivity refers to both the number and relative abundance of timber species, or the species composition of the offtake of a given stand (anywhere along the gradient between high-value, slow-growing hardwood species and low-value, fast-growing, soft wooded species) as identified by individual loggers. We assume that loggers wish to maximize their timber profits and will only harvest low-value species within a volumetric quota if high-value alternatives are no longer available on their land. Market studies provide only a snapshot in time, so we propose that compositional profiles of species selectivity may be crude but reliable surrogates of the degradation status of local timber stocks along the accessibility gradient of tropical logging frontiers.

The notion that industrial scale timber extraction follows a frontier gradient is not new [36,37]. In the early 1970s, large swathes of primary eastern Amazonian forests remained inaccessible, stumpage values were negative, and fiscal incentives and subsidies were handed out to convert forest to pasture. Early tropical logging frontiers are often characterized by highly selective, mobile, low-efficiency extraction targeting the most valuable species, with high transport costs and unstable property rights. As logging frontiers age, investments into transport, extraction and processing infrastructure are consolidated and high-value timber resources rapidly dwindle. Increasing volumes of timber offtake, which here refers to removal rather than harvest, are then required to maintain profits at much smaller margins.

Yet our understanding of the structure and composition of logged forests beyond the first old-growth harvest remains very limited [38]. Each cutting cycle continuously selects offtakes of high-value, slow-growing hardwood species, which may lead to greater functional homogenization of the remaining woody flora which can have complex and unpredictable ecological consequences on the biodiversity of exploited ecosystems [39]. Significant implementation of sustainable forest management is further compounded by pervasive illegal logging activities, which account for 50–90% of all pantropical native forestry products worth US$30–100 billion yr^–1^ or 10–30% of the global wood trade [40], and competes with lower-impact legal logging. For instance, spectral mixing analysis (using the Normalized Differencing Fraction Index [41]) of logging-induced forest disturbance over 3 years (August 2009 –July 2012) across 358,843 ha of eastern Amazonia indicates that 69.7% of this area was logged illegally by unauthorized (‘predatory’) logging operations [42].

Brazil accounts for 85% of the roundlog production in Latin America/Caribbean region, and the total harvested volume was 30.8 million m^3^ in 2012, overlooking the poorly quantified illegal trade [43]. Brazil is estimated to hold 64% of the world’s total intact forest landscapes, 836 Mha [44], of which 72% of natural (i.e. non-plantation) forests are in Amazonia [45]. Since 2006, the Brazilian Forest Service (SFB) has granted logging concessions in National and State Forests to either companies or local communities through a national bidding process (Law 11.284 of 2006). In 2013, 5.3 Mha were available to new concessions with an additional 4 Mha becoming available in 2014 [46]. The 125 Mha State of Pará (the second largest in Brazil) has experienced the oldest history of logging across Amazonia spanning three centuries, but still retains vast untapped timber stocks in remote unlogged forests. Native timber has become the mainstay of the Pará economy since the first road linking the state to southern Brazil was built, with over 2000 sawmills producing 13 million m^3^ of sawnwood in the early 1990s [47]. In 2009, Pará accounted for 47% of the roundlog production, 44% of the gross timber revenue (~US$1.1 billion), and 45% of all direct and indirect jobs in the wood-related sector in Brazilian Amazonia [48].

Although much of the literature condemns conventional over reduced-impact logging, there are few attempts to understand the overall impact of selective logging on biodiversity [49,50]). For example, the extent to which timber harvests beyond the first cut can be financially profitable and/or ecologically sustainable remains poorly known even in low-damage logging operations. Yet the long-term economic viability of selectively‐logged tropical forests is crucial if they are to persist, thereby avoiding more destructive alternative land-use options.

Here, we examine the historical and environmental determinants of the structure of timber offtake by volume and tree species along variable-aged logging frontiers in eastern Amazonia. We summarize data on legal logging operations across their entire size range, which between 2006 and 2012 were authorized to extract some 17.3 million m^3^ of logwood from an aggregate forest area of 638,679 ha distributed across 824 private and community-based logging concessions. We broadly use the term ‘concessions’ to refer to government-sanctioned authorizations (AUTEFs, see below) to harvest timber from land under private or communal ownership, rather than restricting it to timber resources under the public forest domain (see [51]). On the basis of both geographic and historical variables associated with each logging concession, we then attempt to explain broad patterns of available timber stocks, estimated revenues derived from those stocks, and the timber species composition of residual stands. Finally, we highlight important implications for current legal frameworks governing logging concessions and explore the degree to which the current model may be economically viable and demographically sustainable.

## Methods

### Study areas and AUTEF management plans

Mandatory legal approval of forest management plans within the eastern Amazonian state of Pará must be issued to all timber extraction enterprises, including those in communal lands, small to medium private properties, and largeholdings controlled by logging companies, in the form of a ‘Forest Exploitation Permit’ (Autorização de Exploração Florestal; hereafter, AUTEF). The State Environmental Secretariat of Pará (SEMA) issues AUTEF plans, a legal requirement under both SIMLAM (Brazilian Integrated Environmental Licencing and Monitoring System) and SISFLORA (Forest Product Trade and Transport System) for planned timber harvests of any forest site at any spatial scale. We extracted and digitized data from a total of 824 AUTEF plans across Pará sanctioned between 2006 and 2012. These included the name of the rural entrepreneur, community, landholder, or company carrying out each logging operation, the municipal county, and the geographic coordinates of each concession. Of these, a more detailed subset of 446 AUTEF plans (issued between 2009–2012) also included the forest type (planted or natural), the total authorized volume (m^3^) of inventoried timber per tree species per concession to be extracted (minimum cutting diameter at breast height of 50 cm, Normative Ruling no. 05 of 2006), the total standing volume authorized for extraction, the total landholding size, the net size of areas authorized for logging (excluding legally protected riparian forest set-asides where logging is not permitted), and additional set-aside areas within the landholding defined as ‘Legal Reserves’ according to the Brazilian Forest Bill (No. 12.727, http://www.planalto.gov.br/ccivil_03/_Ato2011-2014/2012/Lei/L12727.htm). The remaining 378 AUTEF plans contained all site-specific data in addition to an estimate of total timber volume removed, but did not provide detailed timber species data from local forest inventories. We therefore use either one of these data sets depending on the nature of the analysis. Because timber species were identified *in situ* within concession areas by experienced tree parataxonomists hired to support management plans, we converted vernacular names into their corresponding Latin nomenclature and then removed species-level synonymia whenever necessary based on a comprehensive checklist of timber species of central and eastern Amazonia compiled from multiple sources [52–57].

AUTEF plans granted for exotic tree monocultures, which included eucalyptus (*Eucalyptus*), teak (*Tectona*), and pine (*Pinus*) plantations, were excluded from the analyses. Although Paricá (S*hizolobium amazonicum*) plantations were reported in landholdings (20%), this species is native to Amazonia and was therefore retained in those AUTEF plans defined as ‘natural’ forests. All AUTEF applications to SEMA referred to a unique forest stand of known size based on GPS fixes of property boundaries, although a few exceptionally large landholdings controlled by a logging company may have included more than one AUTEF for different logging compartments exploited in different years (UPAs, Annual Production Units).

### Timber price data

Given that many species accrue significant value along different supply chains and export market prices are affected by complex international demands, we used regional scale logwood prices per timber species in Brazilian Reais (R$ per m^3^ of lumber) available from an official source for the state of Pará that serves as a benchmark for timber merchants [34]. This reflects the dominant domestic market, which consumed 95% of all timber produced in Brazil in 2011 [43], and best reflects realistic transaction prices of unprocessed timber expected by loggers at sawmills or other points of timber sales. Because species-specific timber price data were available for the year 2010, we did not correct those data for inflation because this matches the timing of most of the forest management plans examined here (81%). Timber prices (R$/m^3^) are grouped by DOEPA (2010) into four categories, with gradually fewer timber species commercialized under increasingly higher price brackets: Class A (11 species, 6 genera): > R$75.0/m^3^; Class B (18 species, 12 genera): R$45.0/m^3^—R$74.0/m^3^; Class C (40 species, 31 genera): R$25.0/m^3^—R$44.0/m^3^; and Class D (all other 245 species within 157 genera): R$1.0/m^3^—R$24.0/m^3^. The logwood price data we used are deliberately conservative compared to other sources, which may take into account valuation along supply chains [37,58].

Data on local timber extraction costs were unavailable so our analyses are based on estimates of gross expected revenues. However, extraction costs should scale to the total volumes of timber removed [59] and extent of logging areas exploited, which are taken into account here. Alternative sources of income that may be available to different landholders may also affect local economies of scale and timber species selectivity but are beyond the scope of this study. These may include sales of non-timber forest products and residual dead wood derived from collateral damage at logging clearings (e.g. to meet the high charcoal demand for smelting iron ore in eastern Amazonia), and value-added through timber processing capacities.

### Geographic data

Because exact landholding boundaries of logging concessions were unavailable from AUTEF management plans as spatially explicit polygons, circular buffers of sizes corresponding to each known landholding area (range = 26–844,021 ha), which had been reported in all 824 AUTEF plans, were projected around their geographic coordinates using ESRI ArcMap 10.2.2. Each of these buffers was then assigned an additional 10-km radius external buffer to represent the approximate landscape structure of the forest/non-forest matrix surrounding each AUTEF landholding.

Baseline data on forest structure and composition prior to any large-scale timber extraction were unavailable for logging concession sites. However, we use data from the comprehensive RADAMBRASIL forest inventories (Brasil 1978), which were conducted by the Brazilian government from the late 1960s to the early 1970s to map timber resources across Brazilian Amazonia, to estimate the plot-scale aggregate basal area (BA, m^2^/ha) and wood specific gravity (wood density, g cm^–3^) under pre-logging conditions for each AUTEF site. We considered all tree species inventoried within each timber price bracket. RADAMBRASIL is the most extensive network of forest plots ever undertaken across the entire Brazilian Amazon, and included at least 2,345 one-hectare plots surveyed across the region (<ftp://geoftp.ibge.gov.br/>), within which a total of 128,433 canopy trees ≥ 31.8 cm in diameter at breast height (DBH) [or ≥ 100 cm in circumference at breast height (CBH)] were sampled. This was done using an ordinary krigging interpolation of total forest BA within each timber price bracket from all 1-ha plots. We also used RADAMBRASIL data to test for proportional differences in total basal area (m^2^) and total volumes (m^3^) of high vs low value timber (classes A—B and C–D, respectively) available across the four major logging frontiers of varying histories containing the logging concessions examined here. These frontiers are, from the oldest to the most recently exploited: (1) East Pará, primarily along the Belém-Brasília Highway (BR-010) and the main State Highway of Pará (PA-150); (2) Terra do Meio region, along the Transamazon Highway (BR-230); (3) the Calha Norte region of northwestern Pará; and (4) along the Cuiabá-Santarém Highway (BR-163) of southwestern Pará (see map in S2 Fig).

Approximate dates of logging frontiers follows [48], but these were further validated and refined by accounting for the official onset of any INCRA agrarian settlement within a 75-km buffer of each AUTEF geographic centroid (Table A in S1 File). These government-sanctioned agrarian settlements typically mark the arrival of first settlers into new forest frontiers as they rapidly take advantage of new roads into previously inaccessible areas [60]. In addition, earlier cycles of logging in eastern Amazonia typically occurred within 25 km of major roads [61], so dating of logging frontiers corresponding to each AUTEF site was further verified by accounting for the completion year of all major paved and unpaved roads (or road segments) built in previously remote forest regions based on a comprehensive compilation of historical road-building records (see Table A in S1 File for a full list of explanatory variables).

The proportion of forest and deforested areas (in 2011), natural savannahs (*cerrado*), and water bodies were calculated for both internal landholding projections and external buffers using 30-m resolution data from the Brazilian Space Agency *PRODES* project (Table A in S1 File). Deforestation areas under cloud pixels (for which the deforestation year was unknown) were excluded from any landholding projection, but these amounted to only <3% of all pixels. Road traffic data for 2010, including heavy cargo and passenger vehicles, were obtained for all segments of existing paved and unpaved roads within the state of Pará from Brazil’s Ministry of Transport (Table A in S1 File). Heavily used roads are defined as those used by a daily average of ≥1,000 heavy vehicles (buses and heavy cargo vehicles, including roundlog trucks). Sub-municipal scale human population density (HPD, persons/km^2^) IBGE data, including both rural and urban populations, were extracted for all 8,919 census districts across the 144 municipal counties of the state of Pará (in 2011, [62] Table A in S1 File).

Additional analyses encompassing the entire state of Pará were conducted using a grid of 50km x 50km (2,500 km^2^) cells. Peripheral cells straddling state boundaries were segmented so that only portions contained within Pará were considered in our analysis. Using ArcMap 10.2.2, we quantified for each cell (*N* = 564) the cumulative amount of deforestation by 2012, the proportional representation of forest and non-forest land-cover types, the overall density of paved and unpaved roads (km/km^2^), and the mean HPD from the 2007–2010 IBGE census, in addition to the number of AUTEF sites with centroids within a given cell. We then used a generalized linear model (GLM) with a Poisson error structure to examine the numeric incidence of AUTEF plans within 2,500-km^2^ grid cells across the entire state. In addition to the main fixed terms, we also tested the effects of the interaction between proportional deforestation and road density. A comprehensive list of site, landscape, geographic and socioeconomic variables examined and their sources are available in Table A in S1 File.

### Data analysis

Patterns of timber tree species volumetric abundance and dominance within AUTEF management plans were examined using data from *in situ* forest inventories reported for each logging concession area authorized by SEMA. We examined the correlation structure between species-specific timber market prices (R$/m^3^) and total timber stock sizes (m^3^) quantified within any authorized concession area.

Timber tree species within each concession were rank-ordered in terms of their overall stock value (R$), defined as their total volumetric stock (m^3^) multiplied by the species-specific reported timber price per m^3^ to examine the offtake distribution of species-specific timber values (∑ R$ • m^3^). Using the *vegan* package in R, we then constructed Rank Abundance Distribution (RAD) curves [63] in timber stock values (on a log-scale) to derive the evenness J’ [64] in timber revenues across all co-occurring species exploited at a concession. This provides additional insights into the degree to which loggers could maximize harvesting selectivity by focusing on high-value timber to most efficiently meet their maximum legal quota of 30 m^3^/ha, as required in approved AUTEF plans. Pielou’s J’ evenness could thus be defined as a measure of high-grading of an assemblage of coexisting tree species within a concession area. We selected this evenness measure because it is the most widely used in ecology, and is an excellent species-abundance predictor of species richness in tropical forests [65]. J’ values range from 0.0 to 1.0, with larger values representing more even species distributions in reported volumetric offtakes in relation to market values of timber tree species, or a wider offtake portfolio of timber species by value suggesting lower species selectivity. Conversely, steeper RAD slopes represented by lower J’ values indicate high-grading, or timber revenues disproportionately concentrated on only a few highly profitable timber species.

To examine multivariate patterns of species composition we used Nonmetric Multidimensional Scaling (NMDS) ordination based on the total volumetric abundance of different timber species declared within each AUTEF management plan. Vernacular identification of Amazonian trees is often ambiguous at the species level for several tree morphospecies but sufficiently robust at the genus level [66]. However, identification ambiguity is a lesser problem for the much smaller subset of commercially important timber species identified by experienced parataxonomists in the field. NMDS ordinations thus used an abundance-based (Bray-Curtis) similarity matrix including 153 tree genera surveyed across the 446 concessions for which a timber tree inventory was available, once the raw data had been standardized and sqrt-transformed [67]. Stress values at two-dimensional scaling was 0.23 or lower and ordination scores along the first axis (NMDS_1_) are defined as an additional descriptor of the concession-scale composition of timber species in terms of their declared volumetric abundance.

Timber tree assemblage patterns at the scale of individual stands were further related to geographic and historical variables describing each concession using the BIOENV procedure in Primer version 6.0, using the Spearman rank correlation coefficient (**ρ**). The BEST analysis within BIOENV allows the exploration of environmental variables that best correlate with the dissimilarity patterns observed in biotic assemblages by calculating a rank correlation between the Bray-Curtis dissimilarity matrix [weighed in terms of species-specific timber volumes (m^3^) per logging concession] and the Euclidean distances of the abiotic data [68]. BIOENV results were then tested using a non-parametric Mantel test (RELATE) procedure, which compares the global **ρ** to the distribution of **ρ** under the null hypothesis generated by 999 random permutations. To assess differences in genus-level composition of timber volumes among variable-aged logging frontiers we used analysis of similarities (ANOSIM), which compares within- and between-group variances using the R statistic, which ranges from −1.0 to +1.0 with a value of 0 indicating no difference among groups [68]. The significance is determined by comparing the observed R value to the distribution of R under the null hypothesis of no difference between groups (α = 0.05).

GLMs were constructed to model three response variables associated with the total worth of timber resources per concession: (1) the mean estimated density of gross timber value (R$/ha) per concession from trees available at the time of forest inventories; (2) the timber species J’ evenness in total value by volume; and (3) the first ordination axis (NMDS_1_) describing the broad multivariate patterns of genus-level timber composition. We used a negative binomial error distribution to model the first response variable, and a Gaussian distribution to model the second and third variable. To examine these responses we included covariates that described the size, history (frontier age), geographic features, and the landscape structure of each concession (seeTable A in S1 File).

To test for collinearity among variables, pairwise scatterplots and Pearson correlation coefficients were applied to all covariates, none of which exceeded our 0.6 threshold in pairwise scatterplots. Homoscedasticity assumptions, outliers and influential cases were investigated using standardized residuals and Cook’s distance. Variance inflation factor (VIF) and tolerance statistics (1/VIF (model)) were calculated. Within our GLM models, all VIF values remained below a preselected threshold of 3 [69] and tolerances were well above 0.2 [70]. The assumption of independent errors was tested using the Durbin-Watson test, which were within an acceptable range (1–3: [71]), and overdispersion was not found [72]. Spatial autocorrelation can increase type I errors by introducing biases due to violation of the assumption of independent and evenly distributed residuals [73]. To examine the degree to which AUTEF plans were spatially autocorrelated, Moran’s I correlation coefficients were calculated using the residuals of our three GLMs outlined above. No significant residual autocorrelation was found across our models and this was confirmed by inspection of spatial correlograms in R. For each response variable a global model containing all predictors was first developed and then all candidate models were ranked according to the AIC difference between the lowest AIC model and model *i* (ΔAIC). Where model sets ‘best’ model had a weight of <0.9 and ΔAIC < 2, model averaging was used to estimate coefficients [74]. All analyses were done in R version 2.15.1 (R Development Core Team 2014) and Primer version 6.0.

## Results

In total, 9,568,249 m^3^ of timber representing 314 native tree species were declared for legal offtakes in the state of Pará between 2006 and 2012 from 446 private concessions and community-based logging areas for which the taxonomic composition of timber trees was available. We examined the correlation structure between species-specific timber market prices (R$/m^3^) and total timber stock sizes (m^3^) quantified within any authorized concession area. Pearson correlation values are higher where declared harvests for standing high-value timber are largest, but lower and often negative where loggers target primarily low-value timber. This provides insights into the availability of the most valuable tree species to loggers, although low correlation values could suggest either evidence of local depletion of the most desirable species or deliberate restraint from forest high-grading practices.

Total timber volumes (but not species composition) were also available for a further 378 concessions exploited over the same period. These timber management plans are associated with landholding sizes ranging from 26 to 910,307 ha (mean ± SD = 13,810 ± 71,719 ha, *N* = 824). The proportional area within a landholding boundary authorized for timber extraction was highly variable, ranging from 0.14% to 100% of the total property size (mean ± SD = 49.8 ± 27.3% *N* = 824). Absolute timber offtake per management plan ranged between 77 and 298,612 m^3^ of logwood (mean ± SD = 20,966 ± 27,292 m^3^, *N* = 824) depending primarily on sizes of authorized areas, although reported timber offtake rates per unit area were highly invariant (mean ± SD = 27.2 ± 4.5 m^3^/ha, *N* = 824) and below the legally required maximum quota of 30 m^3^/ha.

Authorized timber extraction encompassed a taxonomic spectrum of 314 tree species representing 153 genera and 38 families. However, the total number of exploited taxa per concession site ranged from only 1 to 70 species (22.3 ± 10.6), 1 to 57 genera (20.9 ± 9.1) and 1 to 27 families (12.8 ± 4.5), suggesting high variance in availability of timber stocks and selectivity. Timber prices per cubic meter ranged from R$ 1/m^3^ to R$86.5/m^3^, although this does not include exceptionally high-value species, such broad-leaf mahogany, because these were not listed in any of the concession forest inventories.

Significant predictors in our GLMs explaining the numeric incidence of AUTEF plans within 2,500-km^2^ grid cells across the entire state of Pará (S1 Fig) included the proportion of deforestation (β = 0.150, *P* < 0.001), paved and unpaved road density (β = 0.264, *P* < 0.001), human population density (β = –0.106, *P* < 0.001), and the interaction between road density and deforestation (β = –0.153, *P* < 0.001).

**Fig 1:**
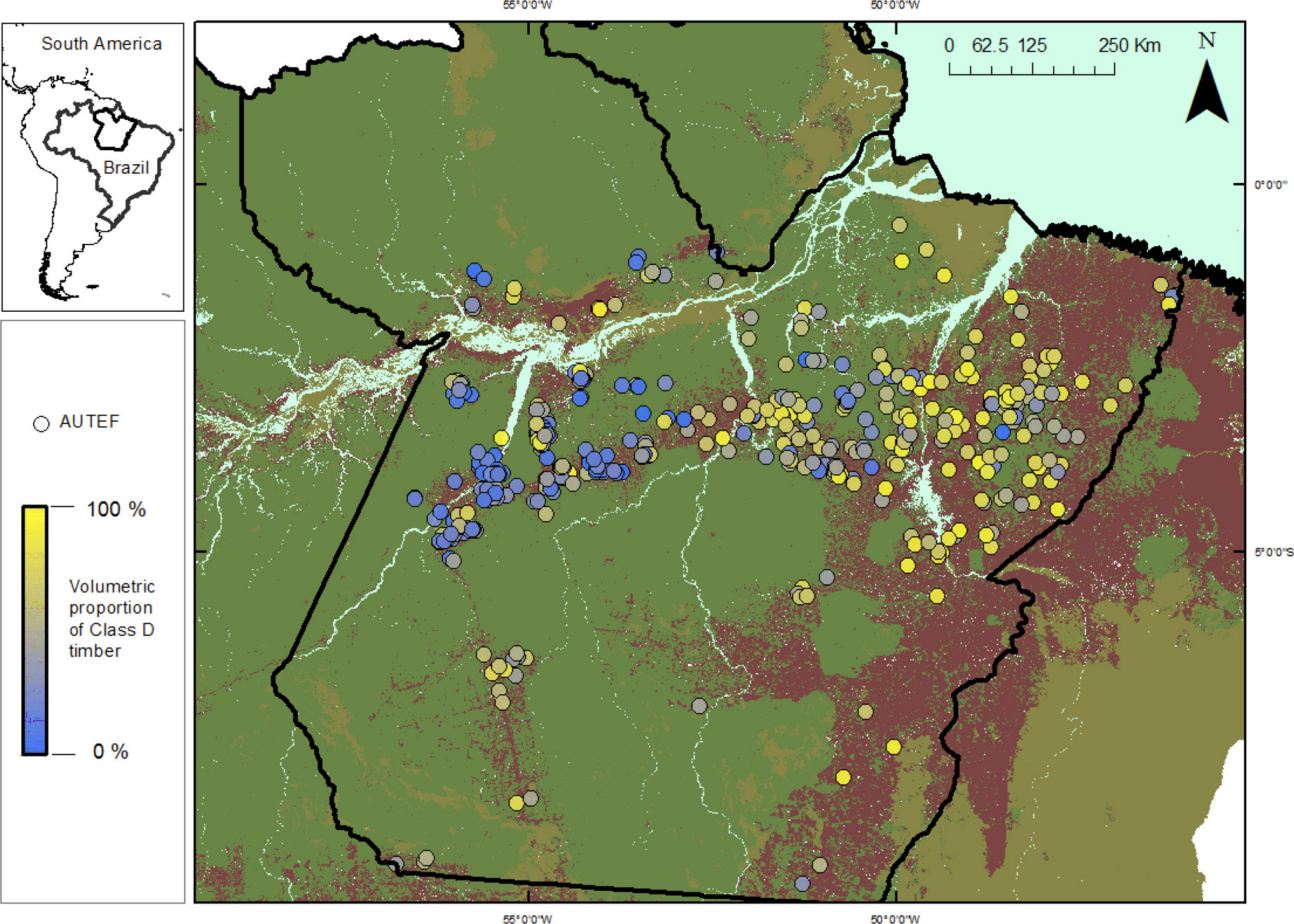
Map of the state of Pará, the second largest in Brazil, showing the geographic distribution of 446 AUTEF management plans for which the species composition of timber stocks were declared based on local forest inventories. Color gradient shows the total fraction of low-value timber species (price class D) by volume (Σ m^3^) declared within each management plan. Standing volumetric stocks of low-value timber species, which comprise the cheapest lumber in local and regional markets, and represent the strongest negative predictor of concession scale logging revenues per unit area. Forest and deforested areas as of 2012 are indicated in green and purple, respectively.

**Fig 2:**
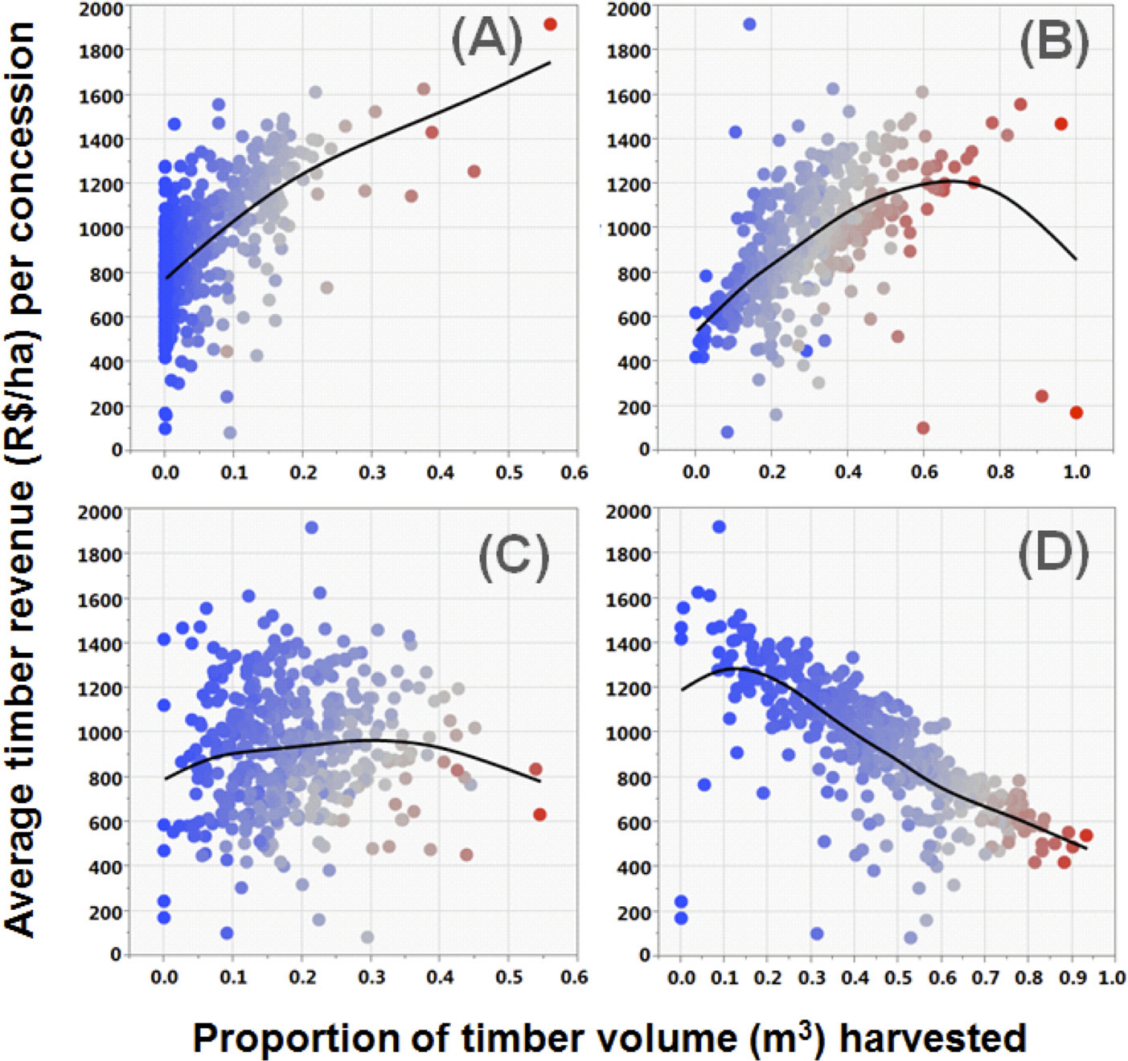
Relationship between volumetric proportions of timber species aggregated by price brackets (classes A, B, C, and D) and mean gross revenues per hectare expected from each authorized concession area. Timber value classes are ordered top to bottom from the highest (A) to the lowest (D). Symbols are colour-coded from low (blue) to high (red) values according to the respective proportion of each price class contributing to the overall timber revenue of each concession site. Solid lines represent a smoother (λ = 0.8) running through all data points.

Considering the 446 AUTEF plans for which species composition was declared from volumetric inventories, the total expected monetary value attributed to each timber price category was 13.2% for the most valuable species (class A), 41.3% for class B, 19.2% for class C and 26.3% for the least valuable species (class D). However, the stand-scale representation of these timber price brackets was highly variable across logging frontiers and concession sites. In general, expected logging revenues per hectare decreased within older logging frontiers in eastern Pará compared to more recent frontiers in western and northwestern Pará, likely reflecting the historical chronological expansion of industrial scale logging throughout the state. This is consistent with the increasingly dominant proportions of low-value timber (class D: <R$24 per m^3^) inventoried at concession sites in the oldest logging frontier (Fig 1). Species composition of timber species harvested by volume clearly affected gross expected timber revenues per hectare derived from each concession, with revenues significantly increasing with proportional offtakes of timber classes A and B, but significantly decreasing with proportional offtakes of timber class D (Fig 2).

**Fig 3:**
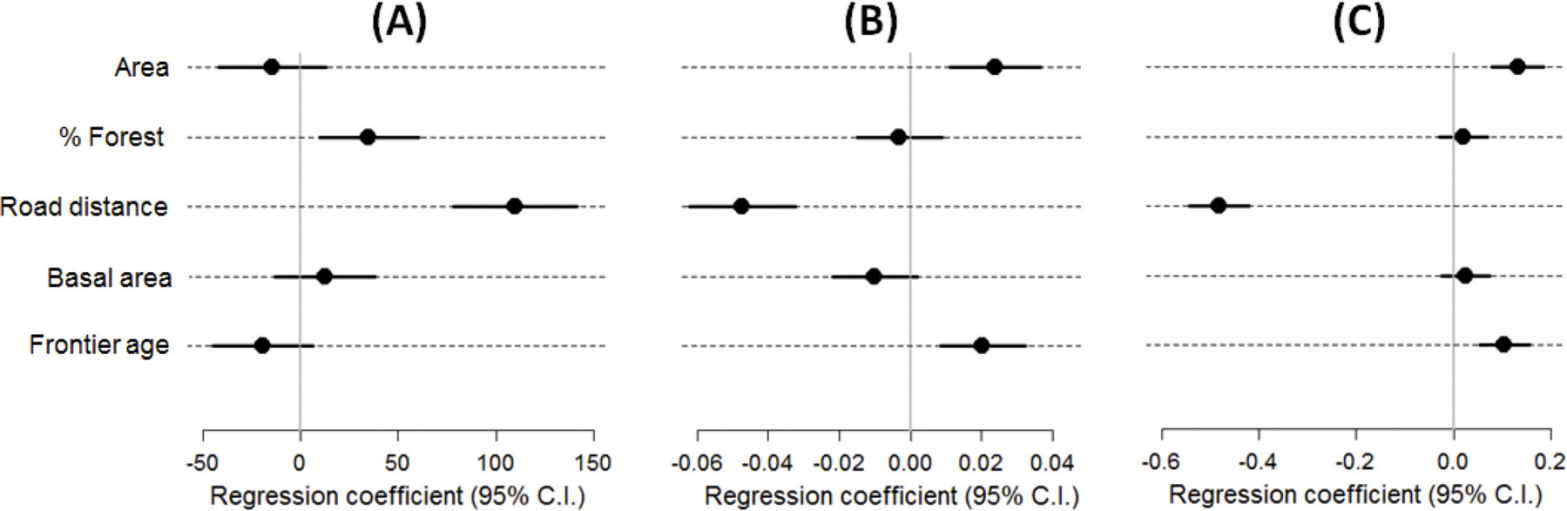
Coefficient estimates (± 95% confidence intervals) showing the magnitude and direction of effects of different forest site and landscape scale variables on timber revenue (R$/ha), species selectivity ranked by timber prices (J’ evenness), and the volumetric genus-level composition of timber offtake (NMDS1). Concession-scale timber revenue, tree species selectivity, and volumetric composition of offtakes were modelled with generalized linear models using the following variables: AREA—net concession areas authorized for timber extraction; % FOREST—percentage of forest cover within a 10-km buffer outside concessions; ROAD DISTANCE—linear distance between each concession and the nearest heavy-traffic paved road; BASAL AREA—predicted pre-logging forest basal area based on an interpolation of 2,345 one-hectare plots from the RADAMBRASIL forest inventory program; and FRONTIER AGE—number of years since the onset of large-scale timber exploitation. Explanatory variables were standardized prior to analyses. For full summary of averaged models, see Table B in S1 File.

**Fig 4:**
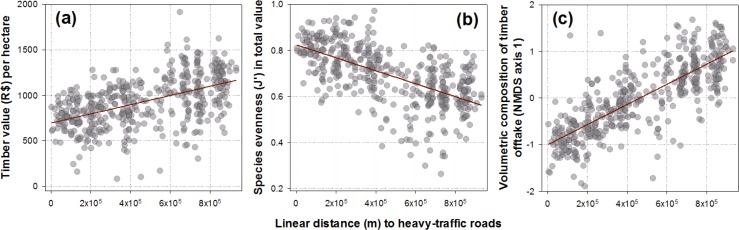
Relationships between linear distances to major access roads and timber revenue (R$/ha), species selectivity ranked by timber prices (J’ evenness), and the volumetric genera composition of timber offtake (NMDS1). Higher revenues per ha (R$/ha) and higher levels of species selectivity (lower Pielou’s J–evenness values) are observed in forest management plans farther away from heavy-use roads. Multivariate patterns of volumetric composition at the genus level (NMDS axis 1), which was inversely correlated with volumetric densities of low-value timber, was strongly positively related to distances from heavy-use roads.

Distance to heavy-traffic paved roads was highly variable across management plans, ranging from 2.49 to 931.7 km (mean ± SD = 464.3 ± 273.2 km). This was the most important predictor consistently appearing in the best candidate and averaged models explaining concession-scale (1) total timber revenue per hectare; (2) volumetric selectivity of tree species (J’) by timber prices; and (3) the first NMDS axis describing multivariate patterns of timber genus composition (Figs 3 and 4). The first NMDS axis was strongly positively correlated with the volumetric density of high-value species (Classes A and B; Pearson r = 0.613, *P* < 0.001, *N* = 446) and strongly inversely correlated with the volumetric density of low-value species (Class D; r = –0.625, *P* < 0.001). Logging concession size, defined in terms of the net authorized harvesting area per landholding, was a significant predictor of both timber selectivity and genus-level composition (Table B in S1 File). Age of logging frontiers was also important in explaining our measure of high grading (expressed as J’ evenness in timber values) and timber genus composition occurring across concession sites (Table B in S1 File). The proportion of forest cover throughout the landscape matrix surrounding each AUTEF landholding was also a positive predictor of timber revenues, suggesting that volumetric densities of high-value species were greater in less deforested areas. Moreover, among all possible combinations of the seven factors tested, distance to major roads and frontier age produced the highest Spearman rank correlations (BEST, ρ > 0.468; *P* = 0.01) with the multivariate structure of timber genera (weighed by volumetric abundance per hectare) inventoried at different concession areas.

**Fig 5:**
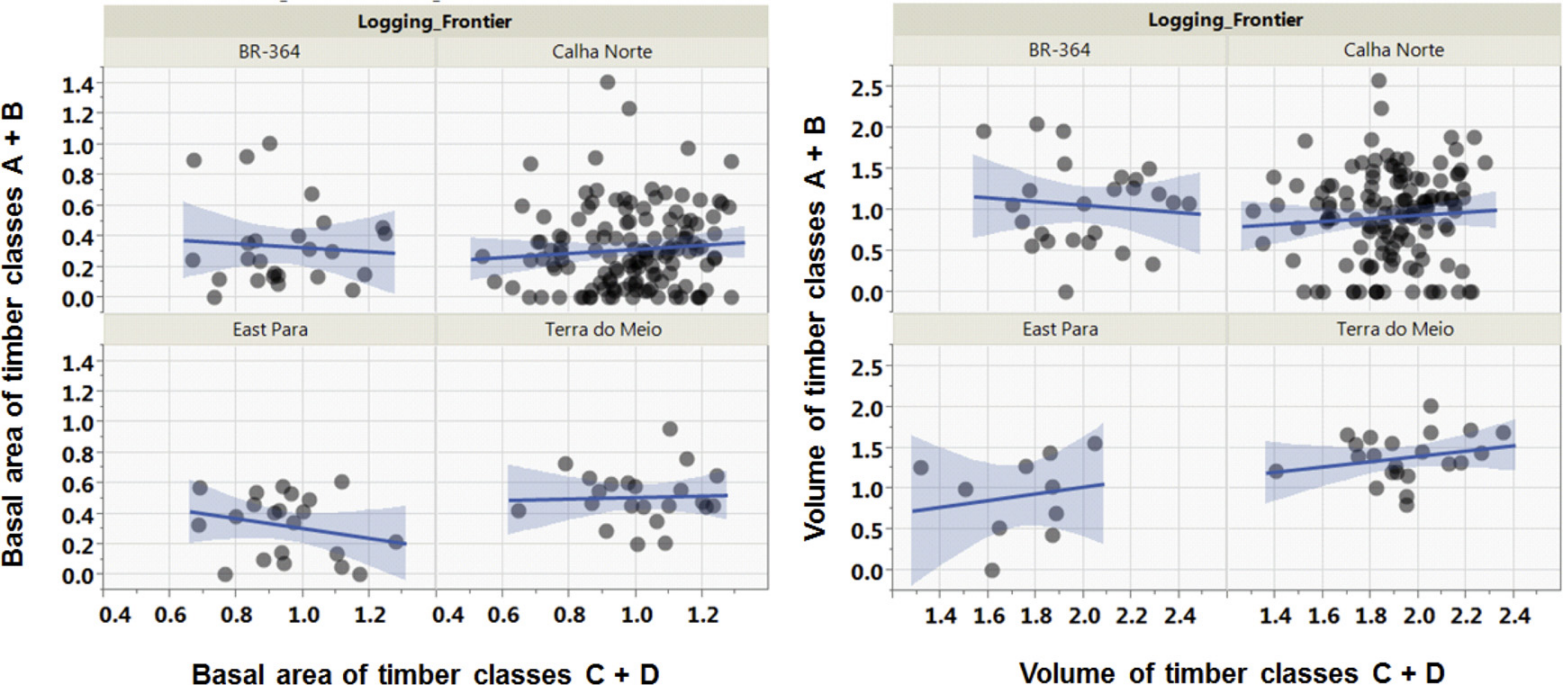
Relationships between the baseline abundance of timber species within contrasting market price classes at four major logging frontiers examined in this study, in terms of the total proportional basal area density (m^2^/ha) and total volume density (m^3^/ha) per 1-ha plot sampled in the early 1970s by the RADAMBRASIL forest inventory program. High-value and low-value timber species are aggregated within price classes A + B and C + D, respectively.

These effects are unlikely to result from baseline differences in the overall abundance of valuable timber species across all concession sites prior to the emergence of mechanized operations in modern logging frontiers. For example, estimates of aggregate forest basal area within canopy tree plots sampled by the RADAMBRASIL forest inventory program yielded no significant effects on total timber revenue, species selectivity, and timber species composition across all of our models. In addition, on the basis of RADAMBRASIL tree plots coinciding with each of our logging frontiers (S2 Fig), we found no significant pre-depletion differences across all four variable-aged logging frontiers in the total basal area (*P* = 0.193) and total timber volume (*P* = 0.311) of high-value timber trees (classes A and B). In fact, historical patterns of timber tree abundance suggest that prior to logging in the 1960s-70s, the most depleted logging frontier (East Pará) had similar relationships between low- and high-value timber species, in terms of both total basal area and total volume, compared to less depleted frontiers (ANCOVAs, *P* > 0.269 in all cases, Fig 5). In addition, there were no baseline differences across logging frontiers in the volumetric abundance of timber species according to timber price classes (S3 Fig). Finally, there were no differences across RADAMBRASIL plots in different logging frontiers in relation to the plot-scale variation in wood specific gravity (wood density) of their canopy trees (S2 Fig), suggesting that the functional composition of currently depleted frontiers included similar relative abundances of heavy-wooded trees compared to less depleted frontiers.

## Discussion

We find no evidence to support the notion that old-growth tropical forest timber stocks across one of the oldest mechanized logging frontiers of lowland Amazonia have been sustainably exploited as a renewable resource capital. Both historical records and the contemporary status of a widespread set of existing forest stands suggest that the most prized and sought after timber species have been repeatedly mined to the point of subregional demographic collapse as a function of local supply/demand conditions mediated by physical access, land-tenure systems, and timber market prices.

Biological resource overexploitation often triggers a pattern of depletion of high-value emblematic species, which results in the sequential targeting of less valuable species locally or displacement of harvesting farther afield into previously undepleted areas where high-value stocks are still abundant [75]. In Brazilian Amazonia, spatiotemporal clustering of logging activity closely tracks the gradual historical expansion of the paved and unpaved road infrastructure, which opens up access into previously unlogged pristine forest areas [17,36]. Depletion of prime tropical hardwood species thus conforms to the general pattern of other extractive industries where multiple species are exploited simultaneously, thereby ensuring that the search effort targeting more abundant species continues to subsidize the exploitation of declining species well beyond their marginal economic value. In the world’s oceans, the largest and most valuable fish are exhausted first before exploitation shifts down trophic levels, sequentially targeting the next biggest fish species available [76]. This is also consistent with historical trajectories of spatial depletion of vertebrate game species by hunters in Neotropical [77] and Afrotropical forests [78]. In both tropical forest and marine ecosystems, populations of fast-growing species increase via different density compensation mechanisms once slow-growing, high-value target species are removed [13,29]. This predicts that lower value species with fast life histories should become increasingly more common in post-depletion markets. Our data clearly supports this sequential depletion pattern for Amazonian timber species. Currently, the most valuable timber species were only available in relatively remote and more recently exploited forest hinterlands, far removed from the oldest logging frontiers, which have been served by heavy-traffic paved roads since the early 1970s, such as the BR-010 and PA-150 Highways in eastern Pará. Timber harvesting cycles under declining supply/demand conditions suggest that concession stands in the oldest frontiers may have been systematically logged twice or three times over the last 45 years. Conversely, relatively undepleted stands in the most recent frontiers, such as Calha Norte and BR-163 Highway, may still retain their full complement of slow-growing, high-value timber trees that are becoming increasingly restricted to unlogged old-growth forests. Our models further indicate that high economic returns per unit area (R$/ha) can only be realized where extensive areas of primary forest are still available in the surrounding landscape, partly because the frontier expansion history of logging and deforestation are inextricably linked [79,80]. Indeed, high levels of timber selectivity, whereby loggers could afford to high-grade timber and prioritize allocation of volumetric offtake to high-value species, were apparently restricted to these areas.

The interplay between large areas of remaining primary forest cover and adequate road access, which results from local deforestation rates and geopolitical allocation of new road-building, largely determines where economically viable timber stocks can be legally extracted. Throughout the state of Pará, AUTEF concessions were largely located in sparsely settled areas where expanding road networks were sufficiently dense, but the extent of cumulative deforestation was still relatively low. Across the entire spectrum of forest loss in eastern Amazonia, this corresponds to areas experiencing intermediate levels of deforestation. For example, nearly one fifth of the state of Pará has been recorded as containing “no timber economic value” [36], but most of this area had either been deforested or completely exhausted of its prime timber resources long before the early 1970s. Given the inter-dependent dynamics of logging and deforestation frontiers in Amazonia, this is a transient and unstable condition because virtually all high road density areas tend to be eventually deforested outside protected areas (e.g. [81]), including Indian Lands, where legally sanctioned logging concessions cannot occur. As logging frontiers grow older in the aftermath of settler occupation, selective timber extraction is gradually forced into ever more remote new logging areas that meet both logistical conditions and financial viability.

Some 90% of all timber species and 67% of the total timber volume (6, 439,474 m^3^) harvested by loggers in eastern Amazonia were of low value, primarily including price classes C and D combined (cf. [82]), but this resulted in only 45.5% of all timber revenues. As expected, the volumetric proportion of the cheapest timber species (class D) in authorized offtakes was inversely related to that of high-value timber species (classes A and B; Fig 2). Baseline estimates of forest basal area of canopy trees (>31.8 cm DBH) based on RADAMBRASIL forest inventories from the late 1960s to early 1970s consistently failed to show significant effects in any of our models, suggesting that patterns uncovered here are indeed a function of more recent depletion of high-value timber, rather than geographic differences in the preharvest price distribution of local timber stocks. This was further supported by additional RADAMBRASIL data as none of our four major regional logging frontiers showed any pre-logging differences in aggregate abundance of high-value timber tree species. In fact, forest plots in the early 1970s in the oldest and most depleted logging frontier (East Pará) on average contained the highest proportion of high-value timber species both in terms of basal area (Σ m^2^/ha) and timber volume (Σ m^3^/ha).

The effects of overexploitation are often exacerbated if overexploited species with slow life histories are also penalized by low abundances, poor long-distance seed dispersal, small geographic ranges, and/or infrequent pulses of seedling recruitment [83]. Other traits that may render timber species vulnerable to population declines induced by overexploitation includes low resprouting capacity after cutting or crushing, and high susceptibility to surface fires due to thin bark [30,84]. Largely unknown nuances restricting optimal reproductive conditions, including the disturbance-dependent episodic recruitment exhibited by mahogany [85] and overharvesting of propagules [18], can render such detrimental consequences even more difficult to predict. Adequate timber regeneration throughout the life cycle of a full complement of tree species also depends on healthy levels of seed dispersal for even the most dispersal-limited large-seeded species, which depends on intact assemblages of large-bodied frugivores (e.g. [86]). Moreover, retaining habitat quality in fully functioning forest ecosystems is crucial beyond post-logging structural damage and compositional changes, and impacts of logging disturbance on forest fauna tend to be more severe in the Neotropics than in the Indomalayan or African tropics [50]. A long-term study at the Tapajós National Forest (Pará) that monitored residual stands prior to and after logging over 30 years indicate that changes in species composition among commercially-valuable canopy trees included average losses of 18 species per treatment area, but no overall decreases in tree species diversity[87].

Our estimates of high-value timber depletion at subregional scales are likely conservative because they take no account of exceptionally valuable timber species, which may have been historically depleted in eastern Amazonia prior to the early-1970s national forest inventories. This includes exceptionally high-value tree species that illustrate chronic population declines, such as broadleaf mahogany (*Swietenia macrophylla*), rosewood *(Aniba rosaeodora*), and moderate-risk Brazil-nut trees (*Bertholletia excelsa*). None of these species were reported in AUTEF concessions because they cannot be legally harvested under Brazilian law, and are officially red-listed in the endangered flora of Brazil (IBAMA 2008, Normative ruling no. 06 of 2008 [88]). However, minor offtakes of rosewood were declared in some AUTEF plans under the vernacular name *pau-rosa* but listed as *Aniba parviflora*, and one concession authorized the offtake of *pau amarelo* (*Euxylophora spp)*. To date, CITES only recognizes the need to control trade of mahogany and rosewood, both of which are listed under Appendix II. At the time AUTEF plans were authorized, Brazilian national forest policy prohibited offtakes of rare species, locally defined as < 0.03 ind. ha^–1^ [i.e. fewer than 3 large trees ≥ 50 cm DBH per 100 ha: Normative Ruling no. 05 of 2006]. Regrettably, this legislation treats tree populations of all species above this density threshold equally, threatening sustainable timber yields (STY) [31]. Recent improvements through Normative Ruling no. 01 of 2015 now requires all concession areas to maintain at least 15% of the large trees or four large trees per 100 ha (per harvested species) within their Annual Production Units, although this is often difficult to ascertain.

Compared to conventional logging, reduced impact logging (RIL) treads lighter on tropical forest structure and composition, and has the potential to be more financially profitable in the long run [59,89]. In practice, however, RIL is far from widely implemented [90,91] and there is wide consensus that RIL alone does not ensure STY [27,29,36,90–96]. In East Africa, a post-logging regeneration time of 50 years is insufficient for forest structure to recover to baseline levels [97], and a minimum cutting cycle of 60 years has been proposed for RIL implementation in the neotropics [98]. This suggests that a second cutting cycle should not occur before ~2030 to achieve STY in many eastern Amazonian subregions, including our oldest logging frontier (East Pará), which was most likely first intensively logged in the early 1970s following the construction of the Belém-Brasília Highway. Our findings further support widespread evidence that a universal cutting cycle is both financially and ecologically unworkable and should be lengthened [38]. Moreover, sustainable forest management practices that can maintain STYs may actually violate prospects of financial profitability, whereas current practices ensure the commercial and demographic depletion of high-value timber species within three harvests in all three major tropical forest regions [99].

AUTEF management plans merely represent a paper commitment of earmarked offtakes that must be fulfilled by law, rather than an accurate record of what timber was actually removed on the ground. The ultimate goal of a landholder is to maximize timber profits within the legal limit of 30 m^3^/ha (Normative Ruling no. 05 of 2006), so that low-value timber species (classes C and D) should be logged only once classes A and B have been exhausted. As such, the management plan is still the most reliable indication of how best to capitalize on timber revenues within a given area. However, we recognize that any observed pattern of exploitation through AUTEF plans is an atypical representation of most logging practices across the Amazon. Species-specific volumetric targets could potentially be met via illegal logging of neighboring forestlands. The disrespect of concession boundaries remains pervasive in Latin America [100], and there are ample opportunities to surreptitiously boost profits with illegally extracted timber from areas outside a nominal licensed concession. The degree to which individual loggers comply with AUTEF guidelines is poorly known, but remote sensing assessments within and outside authorized concession boundaries have identified encouraging improvements. Legally sanctioned logging accounted for a mere 11% of all roundlogs extracted across Pará between 2007–2008, but this gradually improved thereafter to 14% (2008–2009), 35% (2009–2010) and 40% (2010–2011) [101–103]. This represents a significant contribution to urgently needed global efforts to reduce the scale of illegal logging [40]. Unfortunately the proportion of legally sanctioned logging dropped to 22% (73,535 ha) between 2011–2012, following the steady improvement of previous years [42]. The NGO Greenpeace-Brasil, in collaboration with SEMA and Brazil’s Public Prosecutor's Office (MPF: www.mpf.mp.br), carried out a systematic review of all 1,325 extant AUTEF plans in Pará between 2006 and 2013 to assess the extent to which timber laundering occurred [104]. In total, 746 (56.3%) AUTEF plans listed ipê (*Tabebuia serratifolia*) in their inventories and approximately 14% overestimated volumetric offtakes (3,000 m^3^ per concession or 60% above the species average of 2.4 m^3^/ha). Subsequent in situ inspections revealed a plethora of fraudulent activities including illegal timber laundering through illegitimate plans where authorized areas showed no signs of logging. Electronic credit documents (which are issued with AUTEFs and deducted from loggers to credit timber buyers across the chain of custody) were crediting timber well in excess of what had been authorized by management plans before roundlogs were transferred to sawmills exporting timber worldwide.

Between 2000 and 2005 at least 20% of all tropical forests worldwide were selectively logged [105]. In 2012, global scale tropical forest production of roundlogs, sawnwood and plywood combined reached 239.3 million m^3^ [43] and timber extraction from natural forests is likely to expand [82,106]. It is estimates that 4.5 ± 1.35 billion m^3^ of commercial timber is available in Brazilian Amazonia, 1.2 billion m^3^ of which is currently profitable to harvest, resulting in an estimated total stumpage value of US$15.4 billion [107]. As we exhaust Asian and African supplies of tropical hardwoods, market demands on Amazonian timber stocks will only increase. High-income and developed countries are often net wood importers and technocratic solutions aimed at producer-country inefficiencies will be insufficient to meet conservation goals [108]. In meeting these goals a better understanding of the synergies between global timber demands, trade and in-country conservation capacity is crucial [109].

The decisive goal for both biological resource managers and conservation biologists should be to conserve wild species [110]. Effects of logging-induced forest degradation will be further exacerbated by on-going large-scale national development programs designed to meet growing infrastructure and energy demands from an ever-larger human population. A key question is then how can we bring about regional development without compromising conservation goals. This is especially true in light of the national bidding process (Law 11.284/2006) that will legally open access to timber extraction in an additional 4 million hectares of unlogged Amazonian forest from 2014 [46]. To become competitive against illegal logging, low taxes are being lobbied to the Brazilian congress. However, this may flood timber markets, thereby slashing timber prices and forcing law-abiding RIL enterprises out of the market. Many of the current policy failures in managing harvest-sensitive timber stocks in private, communal and public natural forests will thus need to be addressed before the future onslaught of even more widespread timber exploitation.

Unlogged old-growth tropical forests are irreplaceable for biodiversity conservation [111]. However, selective logging can be described as relatively benign compared to alternative forms of tropical forest land use as long as run-away forest degradation can be curbed [20,112,113]. This study provides further evidence that tropical timber tree populations are unwisely exploited as a renewable natural resource in terms of both the forest composition and economic potential of logged forests. Our analysis has important conservation implications, and calls for a better management of the commercial timber offtake portfolio across the entire range of timber species to ensure that high-value species can maintain demographically viable populations. Business-as-usual timber extraction that maximizes short-term profits can lead to loss of natural forest capital for livelihoods along the entire timber supply chain, resulting in severe socioeconomic repercussions in the long run. Even if we overlook the immense but poorly quantified scale of illegal logging, future Amazonian timber supplies are severely threatened by the systemic historical failure in institutional mismanagement, from both federal and state levels, which continues to perform poorly in effective planning, enforcement, and monitoring of sustainable timber yields. Our analyses suggest a rapid rate in population declines in high-value, extinction-prone slow-growing timber species over vast areas, which is unlikely to be easily reversed. We therefore urge national policy makers to curb the largely unchecked tide of widespread depletion of the most harvest-sensitive timber species.

## Supporting Information

S1 Fig Major classes of land cover within the Brazilian state of Pará showing the spatial distribution of 824 private and community-based AUTEF forest management plans approved by SEMA between 2006 and 2012 across a grid of 564 cells of 50km x 50km. The main paved and unpaved roads are indicated in yellow; deforested areas as of 2012 are indicated in orange; non-forest areas refer to natural vegetation types, including Amazonian *cerrados*, outside the closed-canopy forest domain.
(TIFF)

S2 Fig Spatial distribution of 1-ha RADAMBRASIL forest plots inventoried prior to large-scale mechanized logging in the early 1970s. These are color-coded according to the four major eastern Amazonian logging frontier regions examined in this study (left panel: (A) BR-163 Highway: blue; (B) Calha-Norte: red; (C) East Pará: green; and (D) Terra do Meio: purple). Right panel shows the color-coded boxplots describing the mean wood specific gravity (WSG, often referred to as wood density) per canopy tree in those plots, further indicating that the pre-logging WSG profile of trees within different logging frontiers was similar, and that plots in the currently most depleted frontier (East Part) was comparable to less depleted frontiers in their functional profile of canopy tree species.
(TIF)

S3 Fig Nonmetric multidimensional ordination of RADAMBRASIL forest plots based on the volumetric contributions of timber species broken down into different timber price classes (A, B, C and D) across the four major logging frontiers examined in this study. Symbols are color-coded according to logging frontiers: East Pará: red (2) Terra do Meio: orange; Calha Norte: light green; and (4) BR-163 Highway: dark green. There were no significant differences across forest plots grouped by frontiers in the multivariate structure of the abundance of timber species contained according to timber price categories (ANOSIM, 999 permutations, *P* = 0.327).
(TIFF)

S1 File **Table A; Response and explanatory variables considered in this study and their respective data sources. Table B; Final averaged models.** These data sources for Table A are: SEMA/PA: The State Environmental Secretariat of Pará; DOEPA, 2010. N° 31.698 O Diário Oficial do Estado do Pará: Anexo II lista de espécies e definição de categorias com seus respectivos preços individuais e preço médio por categoria, Belem, Brasil.; IBGE, 2011. Instituto Brasileiro de Geografia e Estatística, Available at: http://cidades.ibge.gov.br/xtras/home.php.; INCRA 2013, Instituto Nacional de Colonização e Reforma Agrária, Available at: http://www.incra.gov.br/.; PRODES 2011, INPE, 2012. Projeto PRODES. Monitoramento da Floresta Amazônica Brasileira por Satélite. Available at: http://www.obt.inpe.br/prodes/index.php.; Pereira, D. et al., 2010. Fatos florestais da Amazônia 2010, IMAZON, Belém, PA. PNLT, 2010. Plano Nacional de Logística e Transportes 2010, Ministério dos Transportes, Available at: http://www2.transportes.gov.br/bit/01-inicial/pnlt.html.; RADAMBRASIL forest inventories (Brasil 1978). Available at: /ftp://geoftp.ibge.gov.br/. Table B: averaged coefficient estimates (*β*), unconditional standard errors (SE), P-value, and relative importance (Σ *w*_***i***_) of averaged coefficients calculated over all models retained in the final candidate set for the patterns of timber genus composition (NMDS1), species selectivity (J’) and total estimated timber revenue (R$/ha) from trees available in AUTEF stands across 446 logging concession plans in Pará, Brazil.
(PDF)
