# Supplementary figures and images for: Temporal Decay in Timber Species Composition and Value in Amazonian Logging Concessions
Source: PLoS One. 2016 Jul 13;11(7):e0159035. doi: 10.1371/journal.pone.0159035 (PMC4943729; doi:10.1371/journal.pone.0159035)

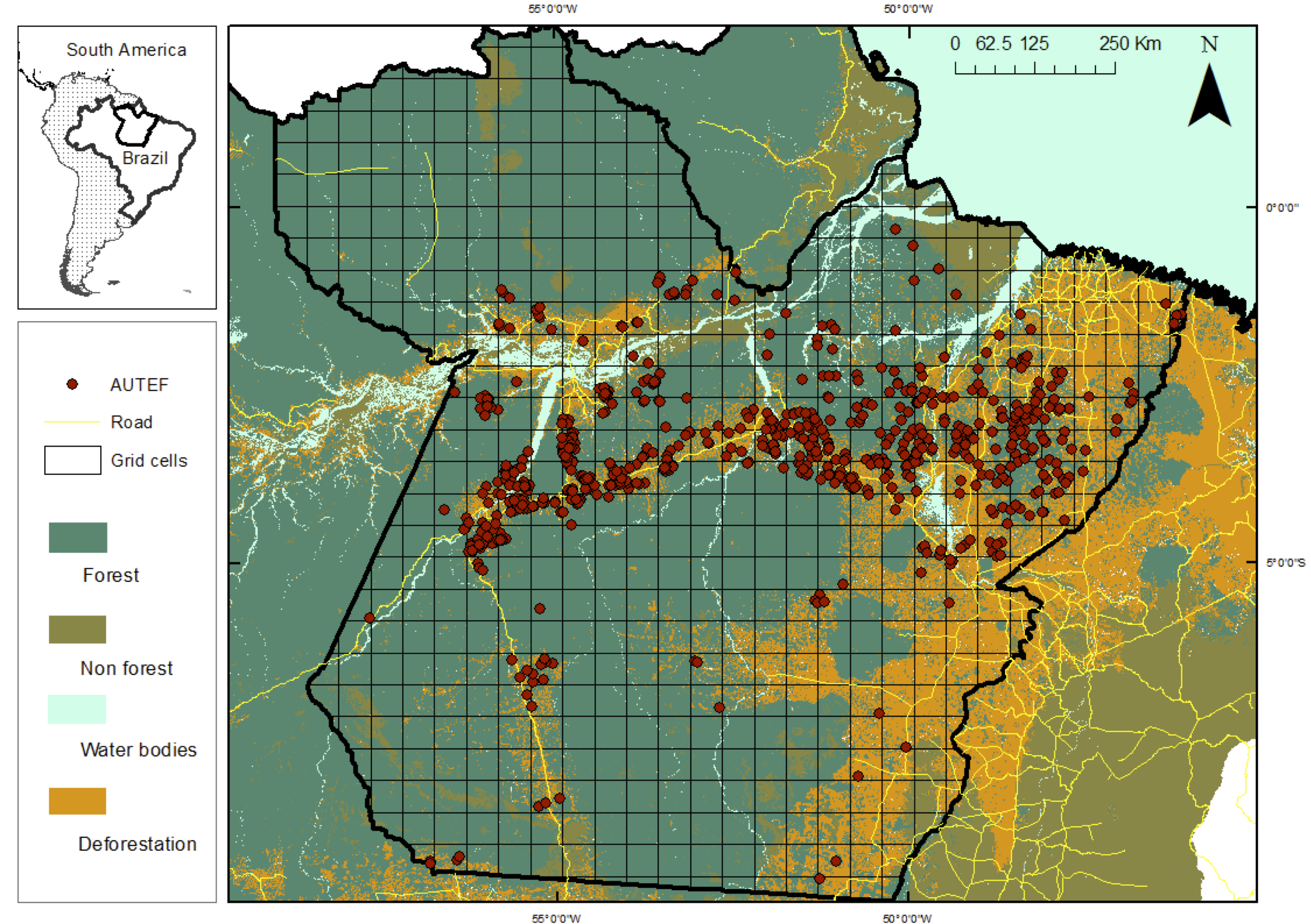

Supplement: S1 Fig — The main paved and unpaved roads are indicated in yellow; deforested areas as of 2012 are indicated in orange; non-forest areas refer to natural vegetation types, including Amazonian cerrados, outside the closed-canopy forest domain. (TIFF) [file pone.0159035.s001.tiff]

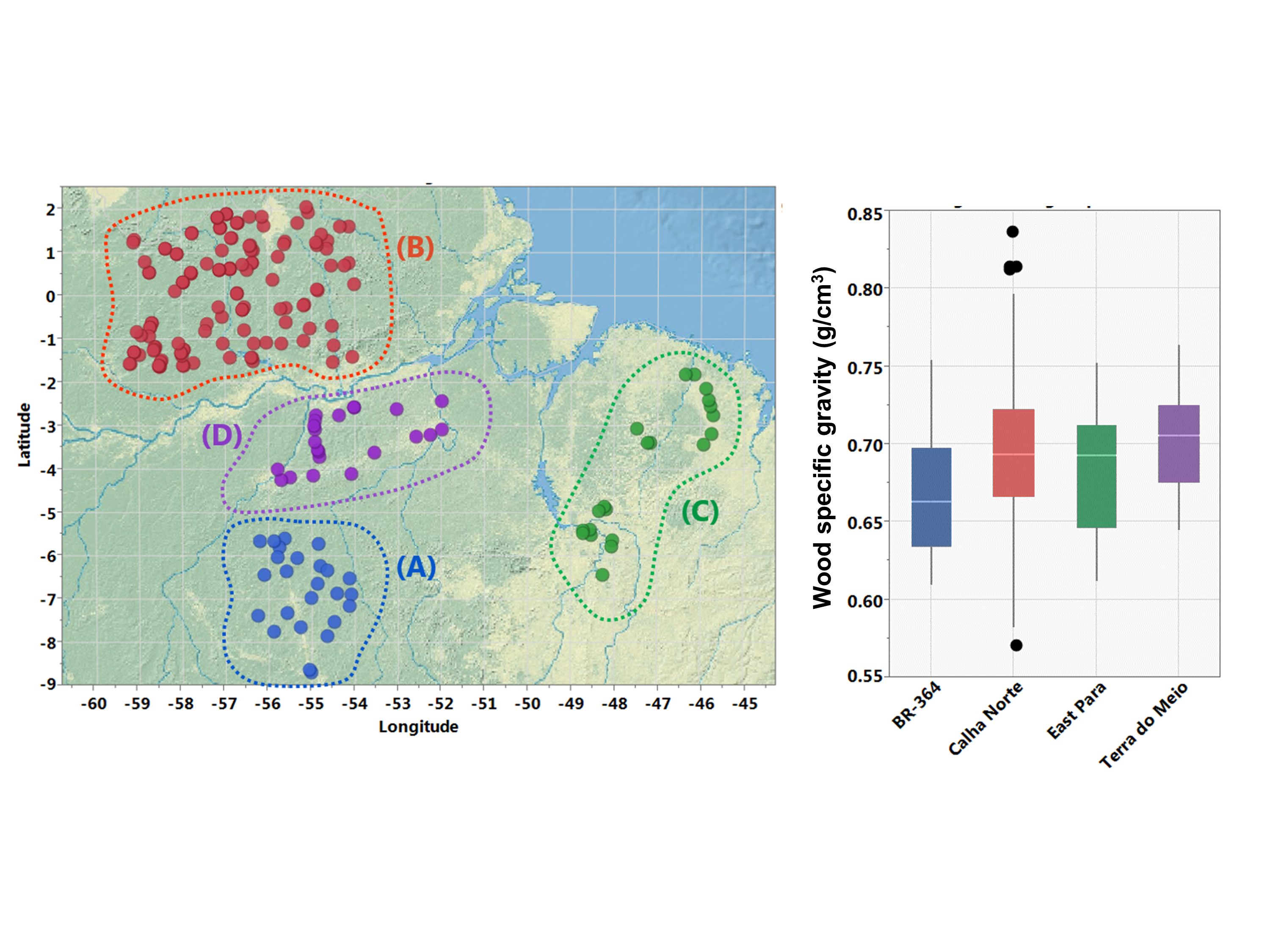

Supplement: S2 Fig — These are color-coded according to the four major eastern Amazonian logging frontier regions examined in this study (left panel: (A) BR-163 Highway: blue; (B) Calha-Norte: red; (C) East Pará: green; and (D) Terra do Meio: purple). Right panel shows the color-coded boxplots describing the mean wood specific gravity (WSG, often referred to as wood density) per canopy tree in those plots, further indicating that the pre-logging WSG profile of trees within different logging frontiers was similar, and that plots in the currently most depleted frontier (East Part) was comparable to less depleted frontiers in their functional profile of canopy tree species. (TIF) [file pone.0159035.s002.tif]

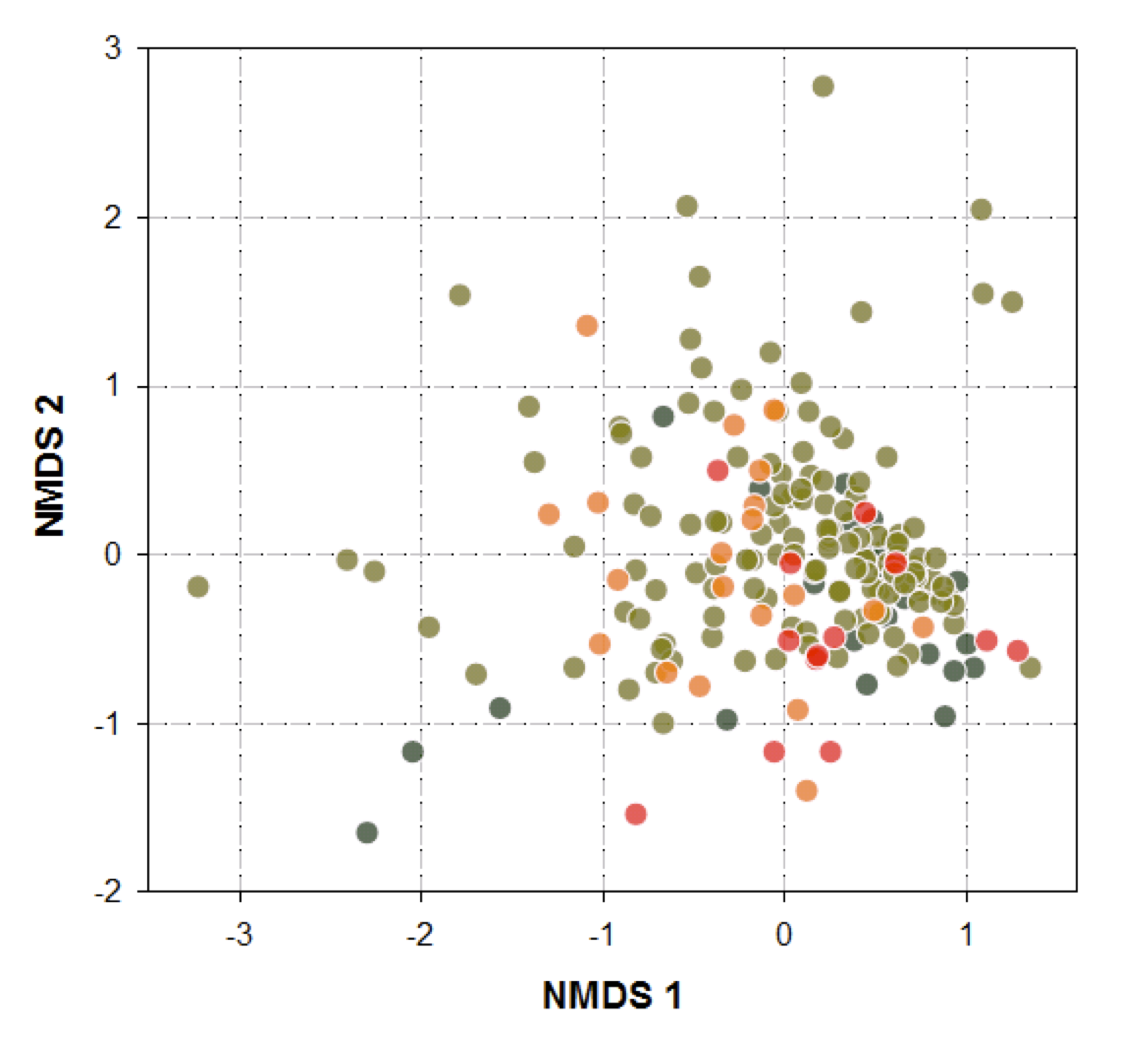

Supplement: S3 Fig — Symbols are color-coded according to logging frontiers: East Pará: red (2) Terra do Meio: orange; Calha Norte: light green; and (4) BR-163 Highway: dark green. There were no significant differences across forest plots grouped by frontiers in the multivariate structure of the abundance of timber species contained according to timber price categories (ANOSIM, 999 permutations, P = 0.327). (TIFF) [file pone.0159035.s003.tiff]
