# Supplementary material for: Temporal Decay in Timber Species Composition and Value in Amazonian Logging Concessions
Source: PLoS One. 2016 Jul 13;11(7):e0159035. doi: 10.1371/journal.pone.0159035 (PMC4943729; doi:10.1371/journal.pone.0159035)
Supplement: S1 File — Table A; Response and explanatory variables considered in this study and their respective data sources. Table B; Final averaged models. These data sources for Table A are: SEMA/PA: The State Environmental Secretariat of Pará; DOEPA, 2010. N° 31.698 O Diário Oficial do Estado do Pará: Anexo II lista de espécies e definição de categorias com seus respectivos preços individuais e preço médio por categoria, Belem, Brasil.; IBGE, 2011. Instituto Brasileiro de Geografia e Estatística, Available at: http://cidades.ibge.gov.br/xtras/home.php.; INCRA 2013, Instituto Nacional de Colonização e Reforma Agrária, Available at: http://www.incra.gov.br/.; PRODES 2011, INPE, 2012. Projeto PRODES. Monitoramento da Floresta Amazônica Brasileira por Satélite. Available at: http://www.obt.inpe.br/prodes/index.php.; Pereira, D. et al., 2010. Fatos florestais da Amazônia 2010, IMAZON, Belém, PA. PNLT, 2010. Plano Nacional de Logística e Transportes 2010, Ministério dos Transportes, Available at: http://www2.transportes.gov.br/bit/01-inicial/pnlt.html.; RADAMBRASIL forest inventories (Brasil 1978). Available at: /ftp://geoftp.ibge.gov.br/. Table B: averaged coefficient estimates (β), unconditional standard errors (SE), P-value, and relative importance (Σ wi) of averaged coefficients calculated over all models retained in the final candidate set for the patterns of timber genus composition (NMDS1), species selectivity (J’) and total estimated timber revenue (R$/ha) from trees available in AUTEF stands across 446 logging concession plans in Pará, Brazil. (PDF) [file pone.0159035.s004.pdf]

S1 File. Table A. Response and explanatory variables considered in this study and their respective data sources. Table B. Final averaged models

Table A

Variable abbreviation	Variable description	Unit	Source
Reais per ha	Total gross revenue expected for the management plan per hectare of net area of authorised land.	R$/ha	SEMA/PA
J-evenness	Pielou's J–evenness of Rank Abundance Distribution curves (by ranked species value) per AUTEF	Numerical	Derived from SEMA/PA using 'Vegan' Package in R
$NMDS_1$	Ordination scores along the first axis of Nonmetric Multidimensional Scaling (NMDS) ordination based on the total volumetric abundance of different timber species as declared in AUTEF plans	Numerical	Derived from SEMA/PA using Primer
Municipio	Municipality the landholding is found in within PA state	Categorical	SEMA/PA
Forest type	Forest type (plantation or natural)	Binary	SEMA/PA
Total reais	Total revenue expected for a given concession. The sum of all species specific values per cubic metre	Brazilian Reais	SEMA/PA and DOEPA 2010
Xlong/ylat	Location of concession centroid	Decimal degrees	SEMA/PA
p.landharvested	Proportion of concession area in relation to total landholding area	Percentage	SEMA/PA
lhsize_ha	Total landholding area	Hectares	SEMA/PA
Concession_area	The authorised area for logging	Hectares	SEMA/PA
Net_concession	Concession area excluding set asides for biodiversity protection under Brazilian law	Hectares	SEMA/PA
vol_perha	Volume of timber offtake per hectare of concession area	Cubic metres per hectare	SEMA/PA
Total_vol	Total volume of timber offtake per AUTEF	Cubic metres	SEMA/PA
no.spplogged	The total number of species logged per AUTEF	Numerical	SEMA/PA
no.genera_logged	The total number of genera logged per AUTEF	Numerical	SEMA/PA
pdeforest	The proportion of deforestation in the AUTEF circular polygon	Percentage	PRODES 2011
pforest	The proportion of forest cover in the AUTEF circular polygon	Percentage	PRODES 2011
pwater	The proportion of water bodies in the AUTEF circular polygon	Percentage	PRODES 2011
pnonforest	The proportion of non-forest in the AUTEF circular polygon	Percentage	PRODES 2011
buffer.pdeforest	The proportion of deforestation in the AUTEF's 10km buffer	Percentage	PRODES 2011
buffer.pforest	The proportion of forest cover in the AUTEF's 10km buffer	Percentage	PRODES 2011
buffer.pwater	The proportion of water features in the AUTEF's 10km buffer	Percentage	PRODES 2011
buffer.pnonforest	The proportion of non-forest in the AUTEF's 10km buffer	Percentage	PRODES 2011
HPD_km	Human population density as a weighted average of the census data within each AUTEF polygon.	Number of inhabitants per squared kilometre	IBGE 2011
buffer_HPD_km	Human population density as a weighted average of the census data within the AUTEF's 10km buffer	Number of inhabitants per squared kilometre	IBGE 2011
Centroid_ndist_AllRds	The AUTEF centroid nearest distance to all extant roads	Metres	IBGE 2011
Centroid_ndist_Heavyrds	The AUTEF centroid nearest distance to heavy-traffic roads. These are defined as those with traffic above and including 1000 heavy vehicles per day	Metres	IBGE 2011
Centroid_ndist_Rivers	The AUTEF centroid nearest distance to all water bodies	Metres	PNLT 2010
Buffer_ndist_AllRds	The AUTEF 10km buffers edge nearest distance to all extant roads	Metres	PNLT 2010
Buffer_ndist_HighFluxRds	The AUTEF 10km buffers edge nearest to heavy traffic roads. These are defined as those with traffic above and including 1000 heavy vehicles per day	Metres	PNLT 2010
Buffer_ndist_Rivers	The AUTEF 10km buffers edge distance to all water bodies	Metres	PNLT 2010
Frontier Age	Logging Frontier Age	Years	INCRA 2013 and Pereira et al. (2010)

**Table B**					
**Predictors**	β	**SE**	**P-value**	**No. models**[a]	Σ ***w_i***
**Timber revenue (R$/ha)** [b]					
Distance to heavy-use roads (km)	4.663e-07	6.129e-08	< 2e-16 ***	7	1.00
Matrix forest cover (%)	1.262e-03	4.852e-04	9.48e-3 **	7	1.00
Frontier age (years)	-2.610e-03	1.349e-03	0.05360	6	0.87
Basal area (m^2)	6.489e-03	5.474e-03	0.23710	2	0.27
Distance to nearest river (m)	4.093e-07	4.688e-07	0.38393	2	0.22
Log10 Concession area (ha)	-2.076e-02	2.590e-02	0.42416	2	0.12
Human population density (/km^2)	1.112e-03	2.791e-03	0.69109	1	0.10
**Species selectivity (J')** [c]					
Log10 Concession area (ha)	3.429e-02	9.858e-03	5.22e-4 ***	3	1.00
Distance to heavy-use roads (km)	-1.930e-07	2.765e-08	< 2e-16 ***	4	1.00
Frontier age (years)	1.257e-03	5.307e-04	01.81e-2 *	4	1.00
Basal area (m^2)	-3.654e-03	2.023e-03	0.071599	3	0.81
Human population density (/km^2)	8.507e-04	1.083e-03	0.433422	1	0.21
Matrix forest cover (%)	-1.176e-04	2.006e-04	0.558849	1	0.18
**Timber species composition (NMDS1)** [d]					
Log10 Concession area (ha)	-2.261e-01	4.052e-02	< 2e-16 ***	6	1.00
Distance to heavy-use roads (km)	1.716e-06	1.065e-07	< 2e-16 ***	6	1.00
Frontier age (years)	-8.129e-03	2.272e-03	3.58e-4 ***	6	1.00
Distance to nearest river (m)	1.688e-06	7.445e-07	0.023733 *	6	1.00
Human population density (/km^2)	-6.313e-03	4.421e-03	0.154420	3	0.50
Matrix forest cover (%)	-7.491e-04	7.641e-04	0.328227	2	0.27
Basal area (m^2)	-7.985e-03	8.089e-03	0.324903	2	0.27

*Significance codes: 0 '***' 0.001 '**' 0.01 '*' 0.05*

[a] Number of models containing each predictor variable over all models retained in the final candidate set.

[b] r-squared estimate from the full model (which equals that of the top model): 0.27

[c] r-squared estimate from the full model (which equals that of the top model): 0.33

[d] r-squared estimate from the full model (which equals that of the top model): 0.66
